# Role and regulation of MKP-1 in airway inflammation

**DOI:** 10.1186/s12931-017-0637-3

**Published:** 2017-08-10

**Authors:** Seyed M. Moosavi, Pavan Prabhala, Alaina J. Ammit

**Affiliations:** 1 School of Life Sciences, University of Technology Sydney, Sydney, NSW Australia; 2 Woolcock Emphysema Centre, Woolcock Institute of Medical Research, University of Sydney, Sydney, NSW Australia; 3 Department of Molecular Pharmacology, University of Groningen, Groningen, The Netherlands; 4 Groningen Research Institute for Asthma and COPD, University Medical Center Groningen, University of Groningen, Groningen, The Netherlands; 5 Groningen Research Institute for Pharmacy, University of Groningen, Groningen, The Netherlands

## Abstract

Mitogen-activated protein kinase (MAPK) phosphatase 1 (MKP-1) is a protein with anti-inflammatory properties and the archetypal member of the dual-specificity phosphatases (DUSPs) family that have emerged over the past decade as playing an instrumental role in the regulation of airway inflammation. Not only does MKP-1 serve a critical role as a negative feedback effector, controlling the extent and duration of pro-inflammatory MAPK signalling in airway cells, upregulation of this endogenous phosphatase has also emerged as being one of the key cellular mechanism responsible for the beneficial actions of clinically-used respiratory medicines, including β_2_-agonists, phosphodiesterase inhibitors and corticosteroids. Herein, we review the role and regulation of MKP-1 in the context of airway inflammation. We initially outline the structure and biochemistry of MKP-1 and summarise the multi-layered molecular mechanisms responsible for MKP-1 production more generally. We then focus in on some of the key in vitro studies in cell types relevant to airway disease that explain how MKP-1 can be regulated in airway inflammation at the transcriptional, post-translation and post-translational level. And finally, we address some of the potential challenges with MKP-1 upregulation that need to be explored further to fully exploit the potential of MKP-1 to repress airway inflammation in chronic respiratory disease.

## Background

Airway inflammation drives pathogenesis in chronic respiratory diseases such as asthma and chronic obstructive pulmonary disease (COPD). The important roles played by mitogen-activated protein kinases (MAPK) superfamily members (ERK (extracellular signal related kinase), JNK (c-Jun N-terminal kinase) and p38 MAPK) in promoting pro-inflammatory pathogenesis and disease progression in these chronic respiratory diseases is well-established (reviewed in [1–3]). Over the past decade or so, many researchers around the world, including our group, have discovered the pivotal role played by the MAPK deactivator, MAPK phosphatase-1 (MKP-1: NCBI official full name - dual specificity phosphatase 1 (DUSP1)) in controlling inflammation. Not only does MKP-1 switch off inflammatory pathways by dephosphorylating MAPK family members at key phosphorylation sites, playing a critical negative feedback and homeostatic function in cellular signalling, it is also one of the significant ways in which respiratory medicines used in asthma and COPD achieve their beneficial effects.

Our review will focus on the role and regulation of MKP-1 in airway inflammation. We will initially outline the structure and biochemistry of MKP-1 and summarise the multi-layered molecular mechanisms responsible for MKP-1 production more generally. We will then focus in on some of the key in vitro studies in cell types relevant to airway disease that explain how MKP-1 is regulated in airway inflammation at the transcriptional, post-transcriptional and post-translational level. We will highlight the critical negative feedback cellular signalling function of MKP-1 and summarise evidence that underscores that upregulation of MKP-1 is an important mechanism of action for respiratory medicines. And finally, to highlight the role played by MKP-1 in the temporal regulation of cytokine expression we will touch on some more recent studies that show that even though MKP-1 might be abundant, it might not be active due to oxidation. These are the future research challenges that need to be understood to fully exploit the potential of harnessing the anti-inflammatory power of MKP-1 to resolve chronic respiratory disease.

## Asthma and COPD are chronic respiratory diseases driven by inflammation

Chronic respiratory diseases such as asthma and COPD are driven by inflammation. Corticosteroids are mainstay anti-inflammatory therapies that are effective in the majority of people with asthma. However, significant proportions of the population with asthma (5-10%) are resistant to corticosteroids and are classified as having severe asthma [4]. Corticosteroid insensitivity and resistance is also prevalent in people with COPD (reviewed in [5]). Chronic inflammation in the lungs of people with COPD drives damage and long-term decline in lung function and, unfortunately, current COPD medications have failed to slow the accelerated rate of lung function decline [6], even when long term studies have been undertaken in asymptomatic subjects with early disease [7, 8]. Thus, there is an urgent need to develop efficacious anti-inflammatories to prevent disease progression. This is where corticosteroids potentially have merit; however, corticosteroids are much less effective in COPD than in asthma due to intrinsic corticosteroid insensitivity that exists in COPD (reviewed in [5, 9]).

Improved anti-inflammatory treatments for chronic respiratory diseases are urgently needed. To achieve this goal, we require an in depth understanding of the molecular mechanisms responsible for repression of airway inflammation. This knowledge is essential to allow design and development of improved and efficacious pharmacotherapeutic strategies for treating and preventing lung function decline in people with chronic lung disease. Upregulation of the endogenous MAPK deactivator, MKP-1, has potential. Hence, to achieve a better understanding of the importance of MKP-1 and its regulatory control of MAPK-driven pro-inflammatory pathways, the general structure and biochemistry of these enzymes will be summarised in next sections.

## MAPK superfamily

MAPKs are protein kinases that transduce extracellular stimuli to different types of cellular responses. Their function and regulation have been conserved throughout evolution from unicellular organisms such as brewers’ yeast, to complex species, including humans (reviewed in [10]). MAPKs are stimulated by different mediators including growth factors (platelet-derived growth factor (PDGF), epidermal growth factor (EGF), and nerve growth factor (NGF)) [11], insulin [12], thrombin [13], angiotensin II [14], phorbol ester-type tumour promoter, Ca^2+^ [15], hydrogen peroxide [16], arachidonic acid [17], oocyte maturation activators [18], osmotic stress [19], UV radiation [20], activators of protein kinase C [21], T-cell antigen stimulator [22] and cytokines, including tumour necrosis factor (TNF) and interleukin 1β (IL-1β) [23]. Some of these MAPK-activating stimuli lead to inflammation in airway disease and have been confirmed experimentally in preclinical models (reviewed in [1–3]).

MAPKs are categorised into three MAPK subfamilies; ERK, JNK and p38 MAPK. All three subfamilies carry the sequence –TXY–, where T and Y are threonine and tyrosine, and X is glutamate in ERK [24], proline or glycine in JNK or p38 MAPK [25, 26]. The essential requirement for mammalian MAPK to become activated is phosphorylation of both of these threonine and tyrosine residues [27]. Since MAPKs are regulated by reversible phosphorylation, deactivation of MAPKs can also occur via dephosphorylation at these residues. This is the role and function of the MAPK phosphatases (MKPs). It is important to note however, that while dual phosphorylation of MAPKs is needed for activation, removal of one or other phosphorylation is sufficient to reduce activity [27]. Moreover, this may be achieved by a number of phosphatases, the majority of which are MKPs (see seminal review [28]).

## MKP-1

MKPs, also officially known as dual specificity phosphatases (DUSPs)**,** are responsible for dephosphorylation/deactivation of MAPKs [29–31]. MKPs dephosphorylate threonine and tyrosine residues which are essential for activation of the MAPKs, as described earlier [11, 26, 27]. In this manner MKPs deactivate MAPK-induced cellular signalling and terminate the kinase cascade. Amongst all MKPs, MKP-1 is the most widely studied and it has been suggested that MKP-1 has the potential to serve as a therapeutic strategy for treatment of diseases driven by inflammation (reviewed in [32]).

### Structure and biochemistry of MKP-1

MKP-1 is the first enzyme and the archetypal member of the MKP/DUSP family. Lau and Nathans (1985) first identified mouse MKP-1 cDNA as an immediate early gene induced by serum through differential hybridization screening of a BALB/c 3 T3 cDNA library [33]. The sequence of mouse MKP-1 cDNA (3CH134) was reported in 1992 [34] and shown to encode a protein of ~40 kilodaltons. The human homolog sequence (*CL100*) was revealed as an oxidative stress-induced tyrosine phosphatase gene [35] and the *DUSP1* (*CL-100*) gene was shown to lie on the long arm of chromosome 5 at a band labelled 35 [36].

**Fig. 1:**
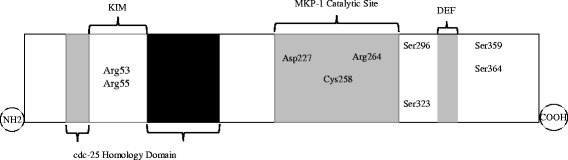
The structure of MKP-1. KIM is located in the NH2 terminus, between two cdc25 homology domains. The catalytic domain is located at the carboxyl terminus. The oxidation of the catalytic Cys258 of MKP-1 protein inactivates its phosphatase activity. In the C-terminus is the DEF docking site for MAPKs, the phosphorylation of Ser359 and Ser364 enhances protein stability, whereas Ser296/Ser323 phosphorylation is involved in the proteasomal degradation of MKP-1

Up to 11 catalytically active MKPs have now been identified in mammalian cells (reviewed by [37]); all MKPs share a common structure comprised of a N-terminal non-catalytic domain and a C-terminal catalytic domain that carries the phosphatase active site sequence (reviewed by [38]). The crystal structure of MKP-1 protein is yet to be resolved; however, due to a high shared sequence identity with other MKPs, such as MKP-2 [39], MKP-3 [40], and MKP-5 [41], the tertiary structure of MKP-1 can be predicted by homology modeling [32]. As shown schematically in Fig. 1, MKP-1 has two cdc-25 homology domains A (CH2A) and B (CH2B) and a conserved protein tyrosine phosphatase (PTP) catalytic site [34, 35, 42, 43]. This conserved PTP catalytic domain conducts dephosphorylation on both threonine and tyrosine residues of MAPKs; hence the name dual specificity phosphatases (DUSPs). As shown in Fig. 1, this site is located in the C-terminus and comprises of three amino acids (Arg264, Asp227 and Cys258, the amino acid numbers correspond with the human MKP-1 sequence); this motif being highly conserved within the MKP family. It is noteworthy that the non-catalytic N-terminal is required for MKP-1 activation [31] contains a region called the **K**inase **A**ctivation **M**otif (KIM), which includes Arg53 and Arg55; two amino acids that are critically important for engaging with MAPKs and catalytic activation of phosphatase function. There is another docking site in MKPs which is involved in initiating phosphorylation of Ser296/Ser323 and inducing protein stabilization [44]. Called “**D**ocking site for **E**RK, **F**XFP” (DEF), DEF and Ser296/Ser323 sites are both important due to their impact on controlling degradation of MKP-1 through ubiquitin-mediated MKP-1 proteolysis [44, 45]. These domains are involved in post-translational regulation of MKP-1.

### Regulation of MAPK superfamily members by MKP-1

Early studies demonstrated that MKP-1 efficaciously dephosphorylates both JNK and p38 MAPK [46, 47]. By titrating MKP-1 expression levels, Franklin et al. suggested that p38 MAPK and JNK are the preferred substrates of MKP-1 [30, 48]. This finding was consistent with that of Dorfman et al. [49] who used MKP-1 knock-out mouse embryonic fibroblasts to show that these cells have no impact on ERK activation during serum stimulation. In contrast, Chu et al. [50] showed that when the expression levels of MKP-1 are high, ERK, JNK and p38 MAPK can be dephosphorylated by MKP-1. Since that time, two decades of evidence clearly show that MKP-1 can dephosphorylate all members of the MAPKs superfamily, although cell type and species selectivity exists (reviewed in [37]).

### MKP-1 expression is regulated at multiple levels

**Fig. 2:**
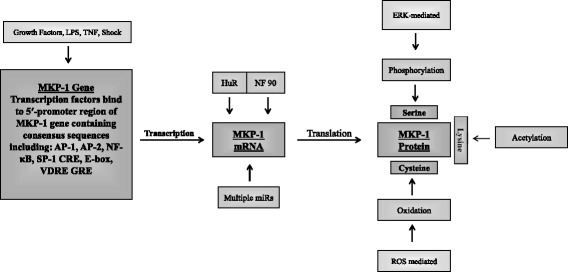
Multi-level regulation of MKP-1 expression. MKP-1 expression is regulated at three levels: transcriptional; post-transcriptional; and the post-translational. Upon extracellular stimulation transcription factors bind to consensus sequences within the MKP-1 5′-promoter region to induce transcription of the MKP-1 gene. Once the gene has been transcribed into mRNA, RNA binding proteins and various micro RNAs (miR) are able to bind to the 3′-untranslated region to modulate the stability of MKP-1 mRNA transcripts. MKP-1 can also be modified at the post-translational level, serines can be phosphorylated, lysines can be acetylated and cysteines can be oxidised, causing MKP-1 protein activity, stability and degradation status to change. See text for abbreviations

Human MKP-1 is a 367 amino acid protein product of an immediate early gene [51] that is localised within the nucleus [52]. MKP-1 expression is regulated at multiple levels; including transcriptional, post-transcriptional and post-translational (as detailed below). These mechanisms allow MKP-1 protein to be rapidly, but transiently, upregulated. As an early example, Kwak et al. [43] stimulated HeLa cells with fetal calf serum and observed a robust increase in the expression level of MKP-1 mRNA after 30 min, which then returns to baseline after 3 h. The transient temporal kinetics profile of MKP-1 is due to the fact that MKP-1 mRNA is post-transcriptionally regulated and MKP-1 protein is degraded by the proteasome. These facts, coupled with the knowledge that MKP-1 expression is regulated at multiple levels by MAPKs themselves (especially p38 MAPK), demonstrates an important negative feedback loop whereby MAPKs regulate MAPK phosphatases, and *visa versa*. Understanding each level of regulation (Fig. 2) offers the potential for pharmacological perturbation.

#### Transcription

A number of consensus binding elements have been demonstrated in the 5′-promoter of MKP-1. In brief, the MKP-1 gene promoter region has been shown to contain binding sites for a number of transcription factors; activator protein 1 (AP-1), activator protein 2 (AP-2), nuclear factor κB (NF-κB), specificity protein (SP-1) and controlled amino acid treatment/binding transcription factor and the cyclic AMP response element (CRE), E-box, vitamin D receptor element (VDRE) and the glucocorticoid responsive element (GRE) [43, 53–58]. Some of these transcriptional regulators have been implicated in MKP-1 upregulation in the context of airway inflammation (the CRE, NF-κB and the GRE in particular) and these will be discussed in subsequent sections.

#### Post-transcriptional regulation

Most of the research on post-transcriptional control of MKP-1 expression has been performed with respect to mRNA stability. This process represents an important regulatory mechanism to control the amount of protein that is translated. mRNA transcripts contain multiple regions within their 3′-untranslated region (3′-UTR) which contain adenosine uridine rich elements (ARE). These *cis*-acting motifs in the ARE regulate mRNA stability and act in concert with *trans*-acting RNA binding proteins to stabilise or destabilise mRNA transcripts. Tristetraprolin (TTP: NCBI official full name – ZFP36 ring finger protein (ZFP36)) is an important RNA binding protein [59] that can destabilise mRNA transcripts of a number of important cytokines in the context of chronic respiratory disease (reviewed in [3]). There is a complex interplay between MKP-1 and TTP that is centred on temporal p38 MAPK phosphorylation. We believe that understanding this regulatory network is critical to allow us to discover how to resolve inflammation in chronic respiratory disease (see later for further discussion). Other RNA binding proteins, such as HuR and NF90, have also been shown to control the stabilisation of MKP-1 mRNA transcripts [60]. There has also been research focusing on microRNAs and the regulation of MKP-1. MicroRNAs (miR) are endogenously expressed non-coding small RNAs that function in a manner similar to *trans-*acting RNA binding proteins, in that they are able to trigger gene silencing and translational repression by binding to the 3′-UTR of target mRNAs [61, 62]. These miRs are numerous and can have conflicting/counter-acting effects on MKP-1. For example, miR-101 was shown to have an inhibitory effect on MKP-1 mRNA in macrophages [63], but miR-708 was shown to augment MKP-1 expression via binding to the 3′-UTR of CD38 in ASM cells [64]. Further research is required to fully reveal the impact of miR on MKP-1 regulation and their role in chronic respiratory disease.

#### Post-translational regulation

MKP-1 activity has been modulated by many types of post-translational regulation. Phosphorylation [65], acetylation [66] and oxidation [67] have all been reported as post-translational modifications of MKP-1 protein.

In regards to phosphorylation, MAPKs themselves impact upon MKPs in a relationship that could be described as somewhat co-dependent: that is, we know that MKPs bind to MAPK and dephosphorylate them at their threonine/tyrosine regions; however, the extent and duration of the effect of MKPs can be dictated by the interaction with MAPKs. This is due to three notable examples of post-translational regulation of the MKP-1 protein. Firstly, there is evidence that the MAPKs need to initially dock at the KIM region found near the N-terminal end of the MKPs, causing MKP proteins to change their conformation and thereby stimulate their phosphatase activity (reviewed in [68, 69]). Secondly, Brondello et al. [44] have shown that there are two carboxyl-terminal serine residues (Ser359/Ser364) on MKP-1 that can be phosphorylated by ERK and alter protein degradation kinetics of MKP-1 causing it to become more stable. Thirdly, ERK was also shown to promote the proteasomal decay of MKP-1. To identify the motif necessary for proteasomal degradation, truncated mutants of MKP-1 were created, which eliminated residues 1–59, effectively removing the KIM domain [45]. It was found that the decay kinetics remained the same, suggesting that the KIM motif was not essential to ERK directed MKP-1 degradation via the ubiquitin proteasome pathway [45, 65]. However, the DEF motif was important and via a series of experiments utilizing point mutations changing key serine residues to alanines it was revealed that ERK-mediated phosphorylation of Ser296 and Ser323 enhanced proteasomal degradation of MKP-1 protein through docking of the Skp1/Cul1/F-box protein Skp2 (SCF^Skp2^) ubiquitin-protein isopeptide E3 ligase [45].

In post-translational protein modifications beyond phosphorylation, Cao et al. [66] reported that MKP-1 protein can be acetylated, and that acetylation of Lys57 within the KIM region of MKP-1 protein enhances interaction of this enzyme with p38 MAPK. This serves to increase MKP-1 phosphatase activity and result in decreased levels of cellular phospho-p38 MAPK and suppression of the MAPK signalling cascade. MKP-1 protein can be also oxidized [67, 70]. This post-translational modification inactivates the enzyme because the catalytic Cys258 within the active site of MKP-1 protein is oxidized. The negative impact of this modification was first demonstrated by Kamata et al. [67], where oxidation at the cysteine residue in the catalytic section of the MKP-1 enzyme induced by reactive oxygen species resulted in protracted JNK activation. Moreover, MAPK-mediated monocyte migration and macrophage recruitment were increased in the presence of oxidised MKP-1 [71] and through S-glutathionylation (the mixed bonds that form between glutathione and cysteine residues in protein), MKP-1 was deactivated as a consequence of redox stress and targeted for proteasomal degradation [72].

## Role and regulation of MKP-1 in airway inflammation

Building on the knowledge of the role, regulation and function of MKP-1 in fundamental cell biology, harnessing the power of the endogenous phosphatase MKP-1 has the potential to control inflammation in chronic respiratory disease. To achieve this, we and others have focused on investigating the mechanisms responsible for MKP-1 expression in clinically relevant models of airway inflammation. Both in vivo and in vitro models have been utilized, but our review aims to bring together the knowledge gained from in vitro models of airway inflammation that predominately use human airway structural cells, primary airway smooth muscle cells and airway epithelia (both primary and transformed cells) in particular. These preclinical research tools have been instrumental in furthering our understanding of the molecular mechanisms regulating MKP-1 production in the context of respiratory disease. A variety of pro-inflammatory stimuli have been used to demonstrate the myriad ways in which MKP-1 expression can be regulated. Moreover, recent studies have also revealed that the mechanism of action for many of the commonly-used respiratory medicines occurs via MKP-1 upregulation. These findings will be outlined in the following sections.

### MKP-1 is a negative feedback effector

Foundational studies that demonstrated the importance of MKP-1 as an anti-inflammatory protein with clinical relevance to airway inflammation came from the arthritis field [73]; MKP-1 was shown to be anti-inflammatory protein responsible for many of the beneficial actions of glucocorticoids (corticosteroids). Most of the early studies in the respiratory field then focused on the ability of corticosteroid-induced MKP-1 to repress cytokine production. Typically, airway cells were pretreated with corticosteroids prior to induction of cytokine production with a range of stimuli. One of the earliest studies was by Issa et al. [74], where ASM cells were stimulated with IL-1β or TNF and the repressive effects of the corticosteroid-induced MKP-1 on production of the chemokine GRO-α assessed (NCBI official full name – C-X-C motif chemokine ligand 1 (CXCL1)). Although the focus of the paper was on the repressive impact of the steroid dexamethasone, this publication was one of the first to show that a pro-inflammatory stimulus (e.g. IL-1β) rapidly (within 1 h), but transiently, induced the production of an anti-inflammatory protein (i.e. MKP-1). We then showed (in 2008 [75]) that TNF also induced MKP-1 by 1 h in ASM cells. Further studies from the Newton lab in Calgary [76] in pulmonary (A549) and bronchial airway epithelial (BEAS-2B) cells then showed that TNF induced MKP-1 protein that peaked at 1 h and returned to basal levels by 2 h (A549) and 6 h (BEAS-2B), respectively. Cytomix (IL-1β, TNF, and interferon γ) also induced the transient upregulation of MKP-1 in A549 cells in the same report.

Since that time much has been learned about homeostatic negative feedback mechanisms exerted by MKP-1. By exploring the molecular mechanisms responsible for MKP-1 protein upregulation by TNF in ASM cells, we found that p38 MAPK is both a stimulus *and* target of MKP-1 [77]. That is, MKP-1 mRNA expression and protein upregulation occurs in a p38 MAPK-dependent manner [77]; however, once MKP-1 protein is expressed, it then acts in a negative feedback manner to dephosphorylate p38 MAPK and reduce expression of p38 MAPK-mediated products (including MKP-1). This is, in part, the molecular basis of transient MKP-1 upregulation and underscores the importance of this MAPK-deactivating phosphatase that serves to limit the extent and duration of MAPK signalling. Clinically, this has been shown in alveolar macrophages from people with severe asthma (corticosteroid resistant) where reduced induction of MKP-1 expression has been correlated with robust activation of p38 MAPK [78].

### Transcriptional regulation of MKP-1

Although a number of transcription factors have been shown to be linked to MKP-1 expression based on the putative *cis*-elements demonstrated in the 5′-UTR region of MKP-1 gene (as outlined above and in Fig. 2), the major ones implicated in MKP-1 upregulation in airway inflammation models in vitro are CRE and GRE. This is not to say that NF-κB-mediated MKP-1 expression is unimportant in this context, rather that investigations have predominately focused on the repressive effects of MKP-1 on NF-κB-mediated cytokine production.

#### CRE

In their MKP-1 promoter analysis, Kwak et al. [43] demonstrated that the putative *cis* elements that might regulate MKP-1 gene expression included two CRE (positions −163 and −118 bp from the transcription start site). CRE is activated by cAMP and numerous publications in the past decade have shown that stimuli that increase cAMP in airway cells increase MKP-1. As elevation of intracellular cAMP in ASM cells is the mechanism of action responsible for the bronchodilatory impact of β_2_-adrenergic agonists on bronchospasm, this CREB-linked transcriptional pathway has important consequences towards understanding the molecular mechanisms responsible for commonly-used respiratory medicines. This unifying, cAMP-dependent, principle links a diverse range of molecules with MKP-1 induction, including: the bioactive sphingolipid found increased in asthmatic airways, sphingosine 1-phosphate (S1P) [79–81]; the cell-permeable cAMP elevating agent dibutyrl cAMP [79]; the adenylate cyclase activator forskolin [79]; short (salbutamol) and long-acting β_2_-agonists (LABAs: formoterol and salmeterol) [82–84]; inhibitors of phosphodiesterase 4 (PDE4) (including cilomilast, rolipram, piclamilast and roflumilast N-oxide), in combination with formoterol [85, 86]; and prostaglandin E_2_ [87]. Research has conclusively shown that these molecules all increase cAMP in airway cells and result in increased MKP-1 production. In some investigations, CREB phosphorylation was also shown [79], and the cAMP-dependent protein kinase A (PKA) pathway was implicated through the use of non-specific pharmacological inhibitors, such as H-89 [83], or more conclusively demonstrated via adenoviral expression of the PKA inhibitor, PKIα [83]. In elegant studies, Kaur et al. [82] have used CRE-reporter constructs in BEAS-2B cells to confirm activation of CRE-dependent transcription in response to cAMP-elevating agents, including β_2_-agonists. This was then linked to upregulation of anti-inflammatory genes, including MKP-1, in A549 and BEAS-2B epithelial cells. BinMahfouz et al. [88] used epithelial cell models with CRE transcriptional activation to test combined PDE3 and PDE4 inhibitors and showed superior efficacy than with either inhibitor alone. Taken together, these studies show that cAMP-elevating agents increase MKP-1.

#### GRE

Researchers in respiratory inflammation have built upon the seminal studies of MKP-1 in the arthritis field, where MKP-1 was first shown to be a novel mediator of glucocorticoid action (reviewed in [73]). Corticosteroid-inducible MKP-1 expression in cells with relevance to airway disease is now a well-established finding first discovered a decade ago. In the influential review [89] Giembycz et al. convincingly argued that understanding the mechanistic basis of the interaction between β_2_-agonists and corticosteroids is the “holy grail” that will drive the development of new optimised pharmacotherapeutics. Summarising the data published in full by Kaur et al. [82], they showed that the clinically-used corticosteroids budesonide and fluticasone induced GRE-dependent transcription in BEAS-2B cells by stably expressing a GRE-reporter construct, and that dexamethasone increased MKP-1 mRNA transcription. Issa et al. [74] were the first to show in ASM cells that dexamethasone induced MKP-1 mRNA and protein expression in ASM cells. We then confirmed that dexamethasone induced MKP-1 protein upregulation in ASM cells [75], as did fluticasone. Dexamethasone also induced MKP-1 in human pulmonary (A549) cells in a temporal manner not too dissimilar to the upregulation profile in human bronchial epithelial cells (BEAS-2B) [76]; establishing this transformed cell line as a valuable model to explore MKP-1 (as confirmed by us in [90, 91]).

However, despite the fact that corticosteroids are known inducers of MKP-1 expression, and steroids are widely accepted as powerful anti-inflammatories, the molecular mechanisms responsible for their actions, and the role played by MKP-1, has been the subject of intensive investigation. A complete discussion is outside the scope of this review, thus the reader is recommended several excellent reviews on this subject [92–96]. The classical glucocorticoid responsive element (GRE) is 15 base pairs. Interrogation of the MKP-1 5′-promoter region by Tchen et al. [57] revealed the existence of unusual, relaxed 10 bp *cis*-acting element responsible for steroid induction of the transcriptional promoter. To explore this in the context of airway inflammation, we conducted a sequence of MKP-1 gene promoter analyses where we transfected ASM cells with a luciferase reporter vector containing an ∼3 kb fragment of the human MKP-1 gene promoter upstream of the transcription initiation site (−2975 to +247 bp) and a series of 5′-promoter deletion constructs (kindly provided by Professor Sam Okret (Karolinska Institutet, Sweden) [97]). We showed that dexamethasone activates MKP-1 transcription in ASM cells via a corticosteroid-responsive region located between −1380 and −1266 bp of the human MKP-1 promoter [83]. Notably, this is the region that contains the relaxed GRE consensus sequence identified by Tchen et al. [57]. Thus, for the first time, our study [83] revealed the molecular mechanism responsible for corticosteroid-induced MKP-1 in primary airway cells with direct relevance to inflammation in chronic respiratory disease.

### Post-transcriptional regulation of MKP-1

It is fair to say that most research on MKP-1 mRNA expression in airway inflammation models in vitro have focussed on the contribution of transcriptional regulation, rather than post-transcriptional regulation. In 2012, we conducted two studies [77, 83] that explored whether mRNA stability was a contributor to steady state levels of MKP-1 mRNA expression in ASM cells. Firstly, we examined whether the β_2_-agonist formoterol, or the corticosteroid dexamethasone, regulate MKP-1 mRNA expression via post-transcriptional mechanisms in ASM cells [83]. Using actinomycin D chase experiments and real-time RT-PCR to measure MKP-1 mRNA degradation over time to determine the kinetics of decay, we showed that the rate of mRNA decay was not affected by either agent [83]; supporting a role for increased transcription as the predominant mechanism of action responsible for increased MKP-1 mRNA expression in ASM cells. Secondly, we examined whether TNF increases MKP-1 expression by enhancing mRNA stability in a p38 MAPK-dependent manner [77]. This was important because p38 MAPK is known to post-transcriptionally regulate many important genes, especially MKP-1 [98]. We pretreated ASM cells with vehicle or the p38 MAPK inhibitor SB203580 for 30 min, prior to stimulation with TNF for 1 h and then performed an actinomycin D chase experiment to measure MKP-1 mRNA stability. Intriguingly, we discovered that the p38 MAPK pathway exerts a small, but significant, level of control on TNF-induced MKP-1 post-transcriptional regulation in a precise temporal manner. Since that time, the critical importance of temporal regulation of MKP-1 (and the central role played by p38 MAPK) towards the repression of pro-inflammatory cytokine production has been revealed. This will be discussed further in following sections.

### Post-translational regulation

Two post-translational regulation mechanisms with importance in airway inflammation are phosphorylations (that control proteasomal degradation) and oxidation. Proteasomal degradation of MKP-1 in airway cells is the mechanism responsible for the transient expression of MKP-1 protein observed in a number of studies [74–76]. To confirm this, we pretreated ASM cells with the proteasome inhibitor MG-132 and showed that the temporal kinetics of MKP-1 protein upregulation was impacted and sustained production of MKP-1 was evident [99]. Importantly, proteasome inhibition reduces TNF-induced interleukin 6 secretion in a MKP-1 and time-dependent manner. Moreover, cytokine arrays revealed that MG-132 represses multiple cytokines implicated in asthma [99]. These data highlight the potential of blocking proteasomal degradation of MKP-1 as a therapeutic target in respiratory disease. Furthermore, since the DEF motif controls the interaction with the E3 ligase SCF^Skp2^ and proteasomal degradation of MKP-1, we have argued [32] that blocking this interaction using novel small molecules may result in sustained expression of MKP-1 protein.

The final post-translational modification that is of relevance in the context of chronic respiratory disease is the oxidation of MKP-1. Oxidative stress is prevalent in patients who are smokers and those who have COPD (reviewed in [100, 101]). In this highly oxidative environment there are a lot of reactive oxygen species present and oxidation is possible at the cysteine residues of target proteins. MKP-1 has a cysteine located as part of its catalytic triad (see Fig. 1: Cys258) and oxidation reduces MKP-1 activity. Recent publications on the redox regulation of MKP-1 have supported this assertion [71, 91, 102]. Additionally, since MKP-1 is a p38 MAPK deactivator, it follows that oxidation would increase p38 MAPK and the inflammatory cytokines that are triggered as a result. This aligns with reports of increased p38 MAPK in COPD patients and patients who smoke [103]. Oxidation of MKP-1 may also be a contributing mechanism to corticosteroid insensitivity/resistance. We have demonstrated this both in vivo [102] and in vitro [91] where we show that even though MKP-1 was present, it may have been inactive due to oxidation. The impact of oxidation on MKP-1 needs to be reversed in order to maintain anti-inflammatory phosphatase action. Although the impact of oxidative stress on a number of proteins with pro-resolving roles in pathogenesis of COPD are well recognised (e.g. transcriptional corepressor histone deacetylase 2 and sirtuin 1 (reviewed in [101]), the importance of the loss of MKP-1 function due to oxidisation is currently underappreciated and warrants further investigation. It is highly likely that the redox regulation of MKP-1 is linked to resistance to corticosteroid insensitivity/resistance.

## Clinically-used respiratory medicines induce MKP-1 in vitro and in vivo

MKP-1 expression is now recognised as one of the ways that respiratory medicines mediate their anti-inflammatory benefit. This was originally highlighted by Giembycz et al. in 2008 [89] and since that time much has been learned about how PDE inhibitors and LABAs enhance the anti-inflammatory effects of corticosteroids, and the contribution of MKP-1 (reviewed in [104, 105]). While some anti-inflammatory genes are upregulated synergistically, an elegant series of studies by the Newton group in Calgary show that MKP-1 is enhanced in an additive manner [82, 88, 104–107]. We concur: we have only ever observed additive effects on MKP-1 production when we have combined drug classes in our in vitro studies [83, 84]. A number of important clinical studies have also been published by the Newton group that examine the presence of MKP-1 in human tissue and explore the molecular mechanisms responsible for corticosteroid efficacy in vivo. Kelly et al. [108] used biopsy samples from allergen-challenged asthmatic subjects and could not detect MKP-1 expression 10 days post-corticosteroid (budesonide) treatment; however, the negative results may reflect the fact that MKP-1 upregulation may be more involved in the initial effects of the corticosteroid. Leigh et al. [109] have conducted a large scale microarray analysis of biopsy RNA in a randomized, placebo-controlled crossover study where healthy male volunteers inhaled placebo or budesonide; MKP-1 (aka *DUSP1*) was one of the upregulated genes. Collectively, there is a weight of evidence that proves that clinically-used respiratory medicines induce MKP-1 in vitro and in vivo*.*

## Challenges with MKP-1 upregulation: too much of a good thing, it is all in the timing, or is MKP-1 not always anti-inflammatory?

On the weight of the evidence reviewed above, it appears theoretically plausible that we could exploit the knowledge of molecular mechanisms responsible for MKP-1 mRNA expression and protein upregulation to increase MKP-1 in respiratory disease settings to reduce inflammation. However, this may not be as simple a task as it sounds. It is important to take into account the level and activation status of the other players involved in the resolution of inflammation and how this may be regulated by MKP-1. One of the most important proteins involved in the restraint of inflammation is the destabilising mRNA binding protein, TTP. As mentioned earlier, MKP-1 and TTP form a cytokine regulatory network that is controlled by the phosphorylation status of p38 MAPK. The Clark laboratory at the University of Birmingham, UK, has led the way in new discoveries in vivo using mice deficient in MKP-1 (*Dusp1* ^−/−^) and knock-in mice expressing active TTP (*Zfp36aa/aa*) and these studies underscore the importance of the TTP-mediated anti-inflammatory network [110, 111]. In a recent review [59], Clark and Dean provide clarity to the cooperation between TTP and MKP-1 and the importance of p38 MAPK phosphorylation in TTP functionality. In collaboration [112], we have explored this regulatory network in ASM cells and confirmed that precise temporal signalling is necessary to exert TTP-dependent anti-inflammatory control of cytokines implicated in respiratory disease. In brief, we have shown that TTP expression and activity are regulated by p38 MAPK and controlled in a temporally distinct manner by MKP-1 [112]. Thus, when functional, TTP can curtail airway inflammation in manner similar to that described for other chronic diseases driven by inflammation (viz arthritis) [113]. But the timing of MKP-1 upregulation is the key to whether active TTP is present. For example, if MKP-1 suppresses p38 MAPK to the extent that TTP is not expressed at all, theoretically then the consequence might be similar to that described for p38 MAPK inhibitors (reviewed in [114]); where clinical trials have been disappointing perhaps due to the inhibition of anti-inflammatory proteins (such as TTP). Theoretically, this could be seen as “too much of a good thing”; that is, MKP-1 can repressed p38 MAPK to such an extent that the anti-inflammatory proteins are inhibited along with the pro-inflammatory proteins.

We propose that it is all in the timing and that an in depth understanding of temporal regulation of TTP function holds the key to exploiting the potential of MKP-1 in the future. TTP is a very adaptable molecule controlled by phosphorylation on two key serines (Ser52 and Ser178 in the mouse, Ser60 and Ser186 in the human orthologue) [59]. When phosphorylated on these sites, TTP is stable and unable to be degraded by the proteasome, but is inactive and unable to cause mRNA decay. Intriguingly, as noted by Smallie et al. [111] “TTP is most evident when it is least active and most active when it is least evident”. In support, we treated ASM cells with the steroid dexamethasone 1 h after TNF stimulation (so-called “therapeutic strategy”) and showed that MKP-1 decreased p38 MAPK phosphorylation whilst increasing abundance of the unphosphorylated (active form) of TTP. It is this active form that was responsible for cytokine repression. In addition to the references included above, the recent work from Shah et al. [115, 116] in airway epithelium (both primary cells and cell lines) clearly demonstrate the negative feed-forward control of cytokine expression by TTP and the role played by corticosteroid-induced MKP-1. Undoubtedly, the temporal regulation of TTP function and its control by MKP-1 has complexities that are beyond the scope of this review, but temporal regulation is a rich area of current research activity and the reader is alerted to an excellent recent review by Newton et al. on this topic [117].

Finally, there are a number of reports where DUSP1, or MAPK inhibition, is implicated in the up-regulation of inflammatory gene expression (summarised in [117]). The possibility exists that MKP-1 is not always anti-inflammatory, but whether this is due to the intricacies of DUSP1 action and impact of temporal regulation on anti-inflammatory outcome warrants further investigation. It remains a possibility that some of the earlier publications where steroids, DUSP1, or MAPK inhibition were implicated in the up-regulation of inflammatory gene expression could be due to the temporal regulation that controls glucocorticoid and cytokine crosstalk and the feed-back, feed-forward, and co-regulatory interactions that determine repression [117].

## Conclusions

To harness the power of the MAPK deactivator MKP-1 to repress inflammation in chronic respiratory disease we need to learn from the lessons of the past decade of preclinical studies and ensure that all drug discovery programs make sure that: (i) MKP-1 is expressed; (ii) MKP-1 is active (not oxidized); (iii) MKP-1 is upregulated at the correct time. Ensuring that we consider the off switches that resolve inflammation, as well as the on switches that cause inflammation, will lead to novel and advanced pharmacotherapeutic strategies to treat chronic respiratory disease in the future.
